# Bias Disclosures, Complicated Mice and a Digital Diss

**DOI:** 10.1100/tsw.2001.49

**Published:** 2001-05-04

## Abstract

Nature reports first this week on a controversial new study about the lack of author disclosure in journals of possible conflicts of interest that may or may not color research results. In Science, the mouse genome sequence's problematic progress leads the news.

Less than one percent of original research articles contains citations that disclose possible conflicts of interest, cites *Nature*. The new study of studies, published in April's *Science and Engineering Ethics*, found that of the journals with disclosure policies, only one-third contained any revelatory statements. The controversy over whether to publish possible bias-creators like corporate funding comes partly as a result of an investigation published two years ago by *The Journal of the American Medical Association* that suggested rampant bias in drug efficacy findings when studies were backed by pharmaceutical companies, says *Nature*. But what exactly constitutes a conflict of interest? This question is a central feature of the debate, and observers suggest in *Nature* that the wide scope of possible answers may fuel the lack of conflict-of-interest disclosure by scientists. Interestingly, *Nature* journals themselves do not carry disclosure policies and so were excluded from the present study. *Nature's* editor, Phillip Campbell, said that policy will change. *Science* reports on the new study in its number seven spot.

The current mouse genome data, anticipated eagerly by scientists, is neither easily used or free, reports *Science*. Both private Celera Genomics and the public Mouse Sequencing Consortium have been crunching out mouse genetic data in efforts to produce a draft sequence. J. Craig Venter of Celera says he will publish methods, but that the results of the work may only be viewed by those paying to access Celera's database. The private project's lead scientists say that it has cataloged 99 percent of the mouse genome, using three different strains to compile genomic information. Researchers wanting to use the available raw data from the MSC project will find no assembled genome there either, reports *Science*. In response to the scattered chunks of data, the MSC plans a method shift, aimed at organizing the data into functional genome contexts. Not as publicized as the human genome draft, the mouse genome is critical for scientific discovery, and many biomedical researchers may be increasingly frustrated by the lack of access and usefulness of the current data, notes *Science*. Projections for completion of a functional mouse genome range between 2003 and 2005. *Nature* did not cover this story.

Both *Nature* and *Science* reported the new targeting of scientific endeavor by the entertainment industry using the Digital Millenium Copyright Act (DMCA) to slap a study's results away from peer scientists. A consortium saying that it wanted to test a copyright protection system for online entertainment files (that contain items like motion pictures) opened a challenge to scientists worldwide last year, asking them to crack a digital watermark. A research team, led by Edward Felten of Princeton University, did just that last November — in four different ways. But while planning to present findings at an academic conference, the group found themselves threatened by a copyright infrigement lawsuit from the consortium and canceled their talk. Legal professionals and academic administrators voice deep concern over this event in *Nature* and *Science*, worrying that widespread in-kind moves by commercial groups could hamper the dissemination of scientific information critical for scientific discovery.

This Week in Nature and Science, May 4, 2001
© 2001 TheScientificWorld, Inc.
